# Prevalence and molecular characterization of methicillin-resistant *Staphylococcus aureus* with mupirocin, fusidic acid and/or retapamulin resistance

**DOI:** 10.1186/s12866-020-01862-z

**Published:** 2020-06-29

**Authors:** Wenjing Chen, Chunyan He, Han Yang, Wen Shu, Zelin Cui, Rong Tang, Chuanling Zhang, Qingzhong Liu

**Affiliations:** 1 Department of Clinical Laboratory, Shanghai General Hospital, Shanghai Jiaotong University School of Medicine, 100 Haining Rd, Shanghai, 200080 People’s Republic of China; 2 Department of Clinical Laboratory, Xiaoshan Hospital, Hangzhou, Zhejiang Province China

**Keywords:** Methicillin-resistant *Staphylococcus aureus*, Mupirocin, Fusidic acid, Retapamulin, Resistance mechanisms, Genetic characteristics

## Abstract

**Background:**

The data on the prevalence of resistance to mupirocin (MUP), fusidic acid (FA) and retapamulin (RET) in methicillin-resistant *Staphylococcus aureus* (MRSA) from China are still limited. This study aimed to examine these three antibiotics resistance in 1206 MRSA clinical isolates from Eastern China. Phenotypic MUP, FA and RET resistance was determined by minimum inhibitory concentrations (MICs), and genotypic by PCR and DNA sequencing of the *mupA*/*B*, *fusB*-*D*, *cfr*, *vgaA*/*Av*/*A*_*LC*_/B/*C*/E, *lsaA*-*C*/*E* and *salA* and mutations in *ileS*, *fusA*/*E*, *rplC*, and 23S RNA V domain. The genetic characteristics of resistance isolates were conducted by pulsed field gel electrophoresis (PFGE) and multilocus sequence typing (MLST).

**Results:**

Overall MRSA MUP, FA and RET resistance was low (5.1, 1.0 and 0.3%, respectively). *MupA* was the mechanism of high-level MUP resistance. All low-level MUP resistance isolates possessed an equivocal mutation N213D in IleS; of these, 2 reported an additional V588F mutation with an impact on the Rossman fold. FusA mutations, such as L461K, H457Q, H457Y and V90I were the primary FA mechanisms among high-level resistance isolates, most of which also contained *fusC*; however, all low-level resistance strains carried *fusB*. Except *lsaE* gene detected in one isolate, no other resistance mechanisms tested were found among RET-resistant isolates. Additionally, sixteen PFGE types (A-P) were observed, among which type B was the most common (49/76, 64.5%), followed by types E and G (4/76, 5.3% each) and types C and M (3/76, 3.9% each). All resistant strains were divided into 15 ST types by MLST. ST764 (24/76, 31.6%), ST630 (11/76, 14.5%), ST239 (9/76, 11.8%) and ST5 (7/76, 9.2%) were the major types. PFGE type B isolates with the aforementioned STs were mainly found in mupirocin resistant isolates.

**Conclusions:**

MUP, FA and RET exhibited highly activity against the MRSA isolates. Acquired genes and chromosome-borne genes mutations were responsible for MUP and FA resistance; however, the mechanism for some RET-resistant isolates remains to be further elucidated. Also, the surveillance to MUP in MRSA should be strengthened to prevent elevated resistance due to the expansion of clones.

## Background

Methicillin-resistant *Staphylococcus aureus* (MRSA) is a major pathogen responsible for various hospital-acquired and community-associated infectious worldwide [1]. Because of strong resistance to antibiotics, treatment of MRSA infections is challenging in clinical anti-infective therapy, leading to high risk of mortality and expensive medication [1].

Skin and soft tissue infections (SSTIs) constitute the common diseases caused by *S. aureus*, including MRSA [2], which can be treated with topical antibiotics mupirocin (MUP) and fusidic acid (FA) [3]. However, unreasonable long-term use of these drugs leads to the emergence of resistance, which is a significant public health concern [3]. Therefore, novel topical antimicrobial agent retapamulin (RET) is developed for the treatment of *S. aureus* SSTIs [3].

MUP resistance in staphylococcus is divided into two phenotypes: high-level (MuH, minimum inhibitory concentration (MIC) ≥ 512 μg/mL) and low-level (MuL, MIC = 8–256 μg/mL) [3, 4]. The MuH is mediated by gene *mupA* or *mupB*, and the MuL is related to point mutations in the chromosomal isoleucyl-tRNA synthetase gene (*ileS*) [3, 5]. Previous studies showed that mutations in *fusA* or *fusE* in chromosome confer high-level FA resistance (FAH), and acquired *fusB*-*D* genes that mediate low-level resistance (FAL) [3, 6, 7]. RET has been licensed in USA and Europe for the topical treatment of SSTIs caused by methicillin-sensitivity *S. aureus* and *Streptococcus pyogenes* [8]. The RET resistance in *S. aureus* is often mediated by the point mutations of ribosomal protein L3 (encoded by *rplC*) or the 23S rRNA V domain, or efflux pumps VgaA/Av/A_LC_/B/C/E, LsaA-C/E and SalA, or methylation of the 23S rRNA subunit (methylated by methyltransferase encoded by chloramphenicol-florfenicol resistance (*cfr*) gene) [3, 9, 10].

Several previous studies reported the resistance of MUP and FA in Eastern China [11–14]. However, since Eastern China is a region with a vast territory, the antibiotic resistance spectrum and the resistance mechanisms may be diversified in different hospitals. Therefore, the data on the resistance of both drugs in clinical isolates of *S. aureus* are limited. To the best of our knowledge, there is no information on RET resistance in China.

In this study, we determined the prevalence of MUP, FA and RET resistance among MRSA isolates from Shanghai and Zhejiang province in Eastern China, and analyzed the underlying resistance mechanisms. Furthermore, PFGE and MLST analysis were also carried out for the genetic characterization of resistant isolates.

## Results

### Prevalence of MUP, FA and RET resistance

**Fig. 1:**
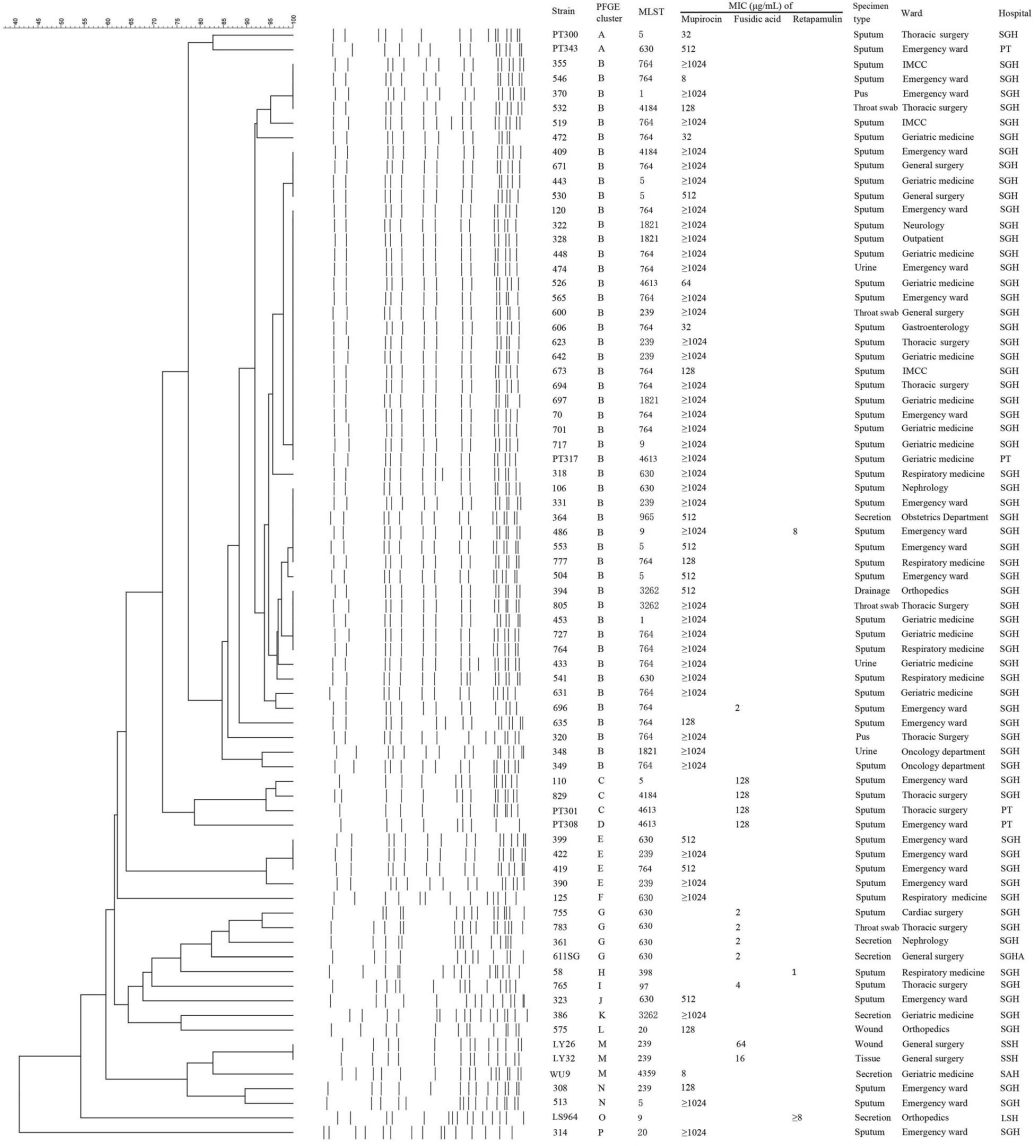
Molecular characteristics, antibiotic MICs and sources of 76 clinical MRSA isolates with MUP, FA and/or RET resistance. The right panel shown the strain number, PFGE types (isolates with > 80% similarity were classified into the same type), MLST results, antibiotics MICs (MUP, FA and RET) and strain sources (including sample type, ward and hospital)

A total of 1206 MRSA isolates were screened using broth microdilution assay, and 49 MuH, 12 MuL, 6 FAH, 6 FAL, 2 RET-resistant and 1 MuH-RET-resistant isolates were identified. The detailed MIC data of the MUP, FA and RET resistance isolates were listed in Fig. 1. Although 75.1% isolates in this collection were obtained from respiratory samples, the resistance rates of these strains to MUP, FA and RET did not differ significantly as compared to those obtained from wound secretion, the second most common specimen type (Table 1).

**Table 1:** Resistance of MRSA isolates from respiratory samples and wound secretion to mupirocin, fusidic acid and retapamulin

Resistance	No. (%) of resistant isolates		χ^2^	*P*
	Respiratory samples (*n* = 906)	Wound secretion (*n* = 200)		
Mupirocin	51 (5.6%)	7 (3.5%)	1.495	0.292
Fusidic Acid	8 (0.9%)	4 (2.0%)	2.170	0.246^a^
Retapamulin	2 (0.2%)	1 (0.5%)	0.540	0.451^a^

^a^, Fisher’s exact test

### Mechanisms of MUP resistance

A total of 49 MuH and 1 MuH-RET resistant isolates contained the *mupA* gene, and no isolates were *mupB* positive (Table 2). The sequences of *Smr*, *Mrm* and *Lmr* DNA fragments were compared to the known *ileS* gene of *S. aureus* (Gene bank accession no. X74219). The findings showed that the 12 MuL isolates possessed N213D mutation in the Smr fragment, and 2 MuL isolates had V588F mutation in the Mrm fragment. No mutations were identified in the Lmr fragment. The mutations were accompanied by different MICs: N213D/V588F, 8 and 32 μg/mL (1 isolate each); N213D, 8 (1 isolate), 32 (2 isolates), 64 (1 isolate) and 128 μg/mL (6 isolates) (Fig. 1 and Table 2). There was not significant difference in the distribution of MIC values between isolates with the single mutation N213D and the double mutation N213D/V588F (*P* = 0.077). In addition, no *mupA* or *mupB* gene was detected in MuL isolates.

**Table 2:** Resistance mechanisms of 76 mupirocin, fusidic acid and/or retapamulin-resistant clinical MRSA isolates

	Mupirocin						Fusidic acid						Retapamulin							
Strain	Phenotype	*mupA*	*mupB*	IleS mutation			Phenotype	Mutation		*fusB*	*fusC*	*fusD*	Phenotype	Mutation		*cfr*	*vagA*/*Av*/*A*_*LC*_/*B*/*C*/*E*	*lsaA*-*C*	*lsaE*	*salA*
				Smr	Mrm	Lmr		Fus A	Fus E					*rplC*	23S rRNA V					
PT300	MuL	–	–	N213D	V588F	–	S						S							
546	MuL	–	–	N213D	–	–	S						S							
532	MuL	–	–	N213D	–	–	S						S							
472	MuL	–	–	N213D	–	–	S						S							
526	MuL	–	–	N213D	–	–	S						S							
606	MuL	–	–	N213D	–	–	S						S							
673	MuL	–	–	N213D	–	–	S						S							
777	MuL	–	–	N213D	–	–	S						S							
635	MuL	–	–	N213D	–	–	S						S							
575	MuL	–	–	N213D	–	–	S						S							
WU9	MuL	–	–	N213D	V588F	–	S						S							
308	MuL	–	–	N213D	–	–	S						S							
110	S						FAH	L461K	–	–	+	–	S							
829	S						FAH	H457Q	–	–	+	–	S							
PT301	S						FAH	L461K	–	–	+	–	S							
PT308	S						FAH	L461K	–	–	+	–	S							
LY26	S						FAH	E8K, V90I, L461K	–	–	–	–	S							
LY32	S						FAH	H457Y	–	–	–	–	S							
696	S						FAL	–	–	+	–	–	S							
755	S						FAL	–	–	+	–	–	S							
783	S						FAL	–	–	+	–	–	S							
361	S						FAL	–	–	+	–	–	S							
611SG	S						FAL	–	–	+	–	–	S							
765	S						FAL	–	–	+	+	–	S							
LS964	S						S						R	–	–	–	–	–	+	–
58	S						S						R	–	–	–	–	–	–	–
486	MuH	+	–				S						R	–	–	–	–	–	–	–
PT343	MuH	+	–				S						S							
355	MuH	+	–				S						S							
370	MuH	+	–				S						S							
519	MuH	+	–				S						S							
409	MuH	+	–				S						S							
671	MuH	+	–				S						S							
443	MuH	+	–				S						S							
530	MuH	+	–				S						S							
120	MuH	+	–				S						S							
322	MuH	+	–				S						S							
328	MuH	+	–				S						S							
448	MuH	+	–				S						S							
474	MuH	+	–				S						S							
565	MuH	+	–				S						S							
600	MuH	+	–				S						S							
623	MuH	+	–				S						S							
642	MuH	+	–				S						S							
694	MuH	+	–				S						S							
697	MuH	+	–				S						S							
70	MuH	+	–				S						S							
701	MuH	+	–				S						S							
717	MuH	+	–				S						S							
PT317	MuH	+					S						S							
318	MuH	+	–				S						S							
106	MuH	+	–				S						S							
331	MuH	+	–				S						S							
364	MuH	+	–				S						S							
553	MuH	+	–				S						S							
504	MuH	+	–				S						S							
394	MuH	+	–				S						S							
805	MuH	+	–				S						S							
453	MuH	+	–				S						S							
727	MuH	+	–				S						S							
764	MuH	+	–				S						S							
433	MuH	+	–				S						S							
541	MuH	+	–				S						S							
631	MuH	+	–				S						S							
320	MuH	+	–				S						S							
348	MuH	+	–				S						S							
349	MuH	+	–				S						S							
399	MuH	+	–				S						S							
422	MuH	+	–				S						S							
419	MuH	+	–				S						S							
390	MuH	+	–				S						S							
125	MuH	+	–				S						S							
323	MuH	+	–				S						S							
386	MuH	+	–				S						S							
513	MuH	+	–				S						S							
314	MuH	+	–				S						S							

*MRSA*, methicillin-resistant *S. aureus*; +, positive; —, negative or no mutation; IleS, isoleucyl-tRNA synthetase; MuH, high-level mupirocin resistance; MuL, low-level mupirocin resistance; FAH, high-level fusidic acid resistance; FAL, low-level fusidic acid resistance; *vagA*/*Av*/*A*_*LC*_/*B*/*C*/*E*, including *vagA*, *vagAv*, *vagA*_*LC*_, *vagB*, *vagC* and *vagE*; *lsaA*-*C*, including *lsaA*, *lsa*B and *lsaC* R, resistance; S, susceptibility

### Mechanisms of FA resistance

To uncover the mechanisms of FA resistance among 6 FAH isolates, the full-length of *fusA* and *fusE* genes were sequenced and compared to those of *S. aureus* ATCC 25923. Herein, we identified 2 isolates that contained H457 missense mutation (H457Q and H457Y, accompanied by MICs of 128 and 16 μg/mL, respectively), while 3 harbored the L461K mutation (MIC = 128 μg/mL), and 1 (MIC = 64 μg/mL) simultaneously possessed L461K, E8K and V90I mutations in FusA (Fig. 1 and Table 2). All the FA resistance isolates were evaluated for *fusB*, *fusC* and *fusD*. Among the 6 FAH isolates, 4 carried the *fusC* gene. Furthermore, all the 6 FAL isolates were *fusB*-positive, and only one carried *fusC* gene (Table 2). No mutations were found in *fusE*, and no isolates were *fusD* positive.

### Mechanism of RET resistance

1/3 isolates with resistance to RET harbored the *lsaE* gene, and the remaining two displayed negative findings for all the resistance mechanisms tested (Fig. 1 and Table 2).

### PFGE

The 76 isolates with MUP, FA and/or RET resistance were divided into 16 patterns: type A-P (Fig. 1). Among 49 MuH alone strains, 40 belonged to type B, 4 were type E, and each of the remaining 5 belonged to types A, F, J, K and N, respectively. One MuH-RET resistant isolate was also type B. Among 12 MuL isolates, 9 were type B, and 3 belonged to type A, M and N, respectively. Type C was the most frequent type in FAH strains (3/6; 50%). Type G was the most common pattern in FAL strains (4/6; 66.7%). Two MRSA resistant to RET alone belonged to types O and H, respectively.

### MLST

Fifteen STs were identified among the 76 isolates studied (Fig. 1). ST764 (24/76, 31.6%) was the most frequent pattern, followed by ST630 (11/76, 14.5%), ST239 (9/76, 11.8%) and ST5 (7/76, 9.2%) and 11 additional STs, namely ST4631 and ST1821 (4/76 each, 5.3%), ST9, ST3262 and ST4184 (3/76 each, 3.9%), ST1 and ST20 (2/76 each, 2.6%), ST965, ST398, ST4359 and ST97 (1/76 each, 1.3%).

## Discussion

MUP is effective for the prevention and treatment of MRSA SSTIs. However, the resistance (including MuL) is beneficial for MRSA treatment and eradication failure [15, 16]. The prevalence rate of MUP resistance in MRSA clinical isolates varies from 0.5–10.1% for MuH and 2.4–8.6% for MuL in USA, 0–75% for MuH and 0–46.7% for MuL in Asia, and from 0.8–98% for MuH and 0–31.2% for MuL in Europe [15]. In the present study, the isolation rates of MuH and MuL were low: 4.1% (50/1206) and 1.0% (12/1206), respectively. Recent studies displayed that the prevalence of MuH is mediated by plasmid-borne *mupA* gene [15], which is the same as our results. Although *mupB*, also a plasmid-borne gene, is correlated with MuH [5], the mechanism is rarely examined in staphylococci, including the isolates investigated in this study. The point mutations in the *ileS* gene, resulting in amino changes in MUP-binding site (located in amino acids 450–650, also named Rossman fold), are the main mechanisms determining MuL [15, 17]. V588F and V631F are well identified frequent mutations in IleS responsible for MuL [15]. In this study, only two MuL isolates (PT300 and wu9) contained the V588F mutation, and no MuL isolates harbored the V631F mutation. Notably, all MuL isolates harbored the N213D mutation that was located in a hotspot amino acid sequence between 200 and 350, as described by Lee et al. [17]. The N213D mutation has been previously reported and are considered to have no impact on the sensitivity of MUP [18]. Although the *mupA* gene located on the chromosome is also associated with MuL [3], we did not detect the gene in our MuL isolates. Also, no other mutations in IleS were found. Lee et al. [17] reported that a mutation of S634F that confers phenotype of susceptibility or MuL in diverse isolates. In view of the above reasons, the contribution of N213D mutation to MuL should be evaluated further.

FA is a steroidal antimicrobial agent that suppresses the production of bacterial proteins by stopping the dissociation of elongation factor G (EF-G) from ribosome [6, 19]. Clinically, the main application of topical FA is for the treatment of SSTIs and decolonization of *S. aureus*, including MRSA; this method is similar to that of MUP [3]. The prevalence of FA resistance reported by recent large studies varies in MRSA isolates from USA (0–0.3%), Australian (4.1–5.1%), Denmark (17.8%), Greece (57.0%) and other European countries (9.9%) [3]. In China, the resistance levels in MRSA are also different in different areas, for example, 3.0–5.3% in MRSA from Beijing, Shanghai, Shenyang and Shenzhen cities [12, 20], and 27.1% in MRSA from Wenzhou city [11]. Compared to the aforementioned data from China, our results showed a very low resistance rate (12/1206, 1.0%).

In *S. aureus*, the mutations in *fusA* (encoding EF-G) or *fusE* (coding for ribosome protein L6, RplF) lead to a decreased affinity of FA for the EF-G ribosome complex [3, 21]. About > 30 point mutations in FusA sequence were described; however, only a few were experimentally verified to play a role in FA resistance [3, 22, 23]. The V90I mutation in domain I (amino acids 1–280) and H457Y, H457Q and L461K mutations in domain III (amino acids 404–480) were observed in this study, and have been previously identified causing FAH in *S. aureus* [21, 22, 24]; also, L461K is the most prevalent mechanism among clinical FAH *S. aureus* strains [3]. L461K also existed in the majority (4/6, 80%) FAH isolates, leading to FA MICs ≥64 μg/mL in the current study. One novel substitution with E8K in domain I was identified in addition to V90I and L461K mutations in the FAH isolate. However, whether the novel mutation is associated with FA resistance is not yet clarified.

Protection of EF-G by FusB family molecules is another mechanism conferring the resistance (low-level) of FA [3]. FusB family proteins (including FusB, FusC, and FusD) can restore the translation of protein by binding to EF-G in the presence of FA [3]. Previous studies showed that *fusB* was the most prevalent in Netherlands and mainland China [11, 12, 25], and *fusC* primarily existed in isolates from Taiwan, Australia, USA and European [26–28]. In our isolates with FA resistance, *fusB* existed in all FAL isolates (6/6), and *fusC* was most prevalent in FAH isolates (4/6, 66.7%). The *fusD* gene was identified in *Staphylococcus saprophyticus*. The gene is related to the “intrinsic resistance of FA” among this species [19]. Hitherto, this determinant is rarely detected in *S. aureus* strains.

RET is a semisynthetic pleuromutilin drug that represses the synthesis of bacterial proteins by interacting with domain V of 50S ribosomal subunit [3]. This drug has a potency to act as an alternative to MUP to eradicate the *S. aureus* colonization, except when used for the treatment of *S. aureus*-induced SSTIs [8, 29]. Currently, limited data are available among clinical *S. aureus* strains worldwide. The resistance rates of 664 UK *S. aureus* (74% are MRSA), 155 USA MRSA, 403 USA MRSA, and 400 USA *S. aureus* from several studies were 0.15, 2.6, 0.25, and 9.5%, respectively [8, 29–31]. In this study, the prevalence of RET resistance was very low (3/1206, 0.24%). In the UK and USA, the RET resistance among *S. aureus* or MRSA with MUP resistance was < 1–2.6% [8, 31]. In the present study, only one MRSA isolate was observed to have resistance to both RET and MUP. For RET resistance in staphylococci, except mutation and/or methylation of ribosomal protein and rRNA, the ABC-F proteins (efflux pumps) encoded by three types of genes (*vga*, *lsa* and *sal*) were alsocritical mechanisms [32]. However, in our 3 RET-resistant isolates, only one was confirmed to contain the *salE* gene. The *salE* gene confers the resistance to pleuromutilins, lincosamides and streptogramin A [32]. Fortunately, the gene was localized on the chromosome or non-conjugative plasmid (pV7073) [32], which limits its transmission among staphylococci. For two strains we could not clarify phenotypic resistance to the detected resistance determinants, implying that there possibly exists other gene or gene variant leading to RET resistance.

ST239 and ST5 are two predominant sequence types in China. However, the strains identified in this study mainly belonged to ST764 (31.6%), which was more than the total percentage of ST239 and ST5 (16/76, 21.1%). Early investigation showed that most of FA-resistant MRSA belong to ST239 and ST5 [7, 27]. However, our findings showed no FA-resistant strains were these two types. ST764 MRSA, first reported in Japan, is a single-locus of ST5 nosocomial MRSA clone with or without the arginine catabolic mobile element (ACME, a feature of CA-MRSA) [33, 34]. In recent years, several studies reported the *S. aureus* clone with ST764 in China [13, 35]. In this study, ST630 (14.5%) was the second most common type, which also became a prevalent *S. aureus* clone causing SSTIs in Wenzhou region, Zhejiang province, China [36]. Figure 1 shows that 66.7% (4/6) FAL isolates belonged to ST630 (PFGE G-ST630), which was similar to the previous report [9]. Notably, 50% (6/12) MUL isolates and 39.0% (16/41) MUH isolates with MIC ≥1024 μg/mL were PFGE B-ST764, and were identified from the same hospital (Shanghai General Hospital) (Fig. 1), indicating clone transmission. In addition, the spread of other MRSA clones with different genetic patterns, such as PFGE B-ST1821, PFGE B-ST239 (4 isolates each), PFGE B-ST5 and PFGE B-ST630 (3 isolates each) were also responsible for the increased MUH in this hospital. It should be noted that some resistant isolates had the same PFGE pattern (such as PFGE B, C, E, G or M), although the ST types were diverse from each other (Fig. 1). These finding hint that these isolates might be clonally related.

## Conclusions

In this study, the MRSA isolates exhibited a low prevalence of resistance to MUP, FA and RET, especially to the latter two, and cross-resistance to the three antibiotics was rare. The *mupA* gene mechanism mediated MuH. The contribution of the N213D mutation in IleS found in our MuL isolates that decreased the resistance of MUP is yet unclear. FusA mutations, FusB and FusC were the frequent genetic mechanisms that mediate FA resistance. Phylogenetic detection showed the transmission of multiple clones, especially PFGE B-ST764 clone that made a major contribution to the increased MUP resistance in this collection of isolates. Owing to the concern of resistance development and clonal dissemination in healthcare, continuous surveillance for the resistance of these topic antibiotics in *S. aureus* is essential in China.

## Methods

### Bacterial isolates

A total of 1206 non-duplicate MRSA isolates from various clinical specimens were collected from 8 hospitals in Shanghai and Zhejiang province, Eastern China (Shanghai General Hospital (1037 isolates from July 2010 to June 2015), Ruijin Hospital (22 isolates during January 2011 to December 2011), Shanghai Sixth People′s Hospital (36 isolates between Decermber 2010 and Decermber 2012), Shanghai People′s Hospital of Putuo District (45 isolates from January 2013 to May 2014), Shuguang Hospital Affiliated to Shanghai University of Traditional Chinese Medicine (21 isolates from February 2014 to September 2014), Shanghai Armed Police Corps Hospital (9 isolates from January 2014 to June 2014), Zhejiang Xiaoshan Hospital (8 isolates from March 2012 to October 2012) and The Central Hospital of Lishui City, Zhejiang Province (28 isolates, July 2013 to September 2014)). The majority of these isolates were obtained from respiratory samples (906/1206, 75.1%) and wound secretion (200/1206, 16.6%). The MRSA isolates were frequently detected in intensive care units, respiratory medicine, geriatric medicine, thoracic surgery and nephrology wards causing maximal impact. All isolates were identified using VITEK microbial identification system (bioMérieux, Marcy l′ Etoile, France). The resistance to methicillin was detected with a 30 μg cefoxitin disk (Oxoid, Basingstoke, UK) [4]. MuH (MIC ≥512 μg/mL), MuL (MIC = 8–256 μg/mL), FAH (MIC ≥8 μg/mL), FAL (MIC = 2–4 μg/mL) and RET resistance (MIC ≥1 μg/mL was defined as resistance in this study) were screened from all the isolates collected by broth microdilution method [4, 6, 14, 37]. For three-antibiotic (MUP, FA and RET) resistant isolates, the methicillin resistance was further confirmed by the amplification of *mecA* and *mecC* genes [35]. MUP, FA and RET were purchased from Shanghai Boyle Chemical Co., Ltd., China. Cation-adjusted Mueller-Hinton broth was produced by Shanghai Comagal Microbial Technology Co., Ltd., China. *S. aureus* ATCC 25923 and ATCC 29213 were used as quality control strains for antibiotic susceptibility testing.

### Total DNA extraction

The cultures of MRSA with MUP, FA and/or RET resistance were incubated with lysostaphin (1 mg/mL) (Sangon Biotech, Shanghai, China) at 37 °C for 0.5 h. Then, the DNA was extracted according to the instructions of the bacterial genomic DNA kit (Tiangen Biotech, Beijing, China), and utilized as a template for PCR assays.

### Detection of MUP, FA, and RET resistance genes

PCR was used to detect the factors mediating MUP (*mupA*, *mupB* and *ileS* (amplifying three fragments of *Smr*, *Mrm* and *Lmr*, which might possess the mutations mediating mupirocin resistance)), FA (*fusA*, *fusB, fusC*, *fusD* and *fusE*) and RET (*rplC*, *cfr*, *vgaA*/*Av*/*A*_*LC*_/*B*/*C*/*E*, *lsaA*-*C*/*E*, *salA* and 23S RNA V) resistance [5, 7–10, 26, 29, 38–41]. The primers and programs for the amplification of genes are presented in Table 3. The DNA sequencing of one randomly selected PCR product for *mupA*, *mupB*, *fusB*-*D*, *cfr*, *vgaA*/*Av*/*A*_*LC*_/*B*/*C*/*E*, *lsaA*-*C*/*E*, and *salA* was used for the identification of target fragments. All the PCR products for *ileS*-*Smr*, *ileS*-*Mrm*, *ileS*-*Lmr*, *fusA*, *fusE*, *rplC* and 23S RNA V were sequenced to determine the putative mutations.

**Table 3:** Primers and PCR conditions for detecting mupirocin, fusidic acid and retapamulin resistance genes in this study

Gene	Primer name	Primer sequence (5′-3′)	PCR amplification program	Size	Reference
Molecular mechanisms related to mupirocin resistance					
*mupA*	*mupA*-F	TATATTATGCGATGGAAGGTTGG	Initial pre-denaturation at 94 °C for 5 min; 30 cycles of denaturation (30 s at 94 °C), annealing (30 s; at 53 °C for *mupA*, and 55 °C for *mupB*, *Smr*, *Mrm* and *Lmr*), and extension (50 s at 72 °C), followed by a final extension step of 7 min at 72 °C	457 bp	5
	*mupA*-R	AATAAAATCAGCTGGAAAGTGTTG			
*mupB*	*mupB*-F	CTAGAAGTCGATTTTGGAGTAG		674 bp	
	*mupB*-R	AGTGTCTAAAATGATAAGACGATC			
*ileS* (including the following 3 fragments)					
*Smr*	*Smr*-F	ATAAAGGTAAAAAGCCAGTTTATTGGT		200 bp	38
	*Smr*-R	TAATCGCAACATTTGATGGAATTGTC			
*Mrm*	*Mrm*-F	TCCCAGCAGATATGTATTTAGAAGGT		450 bp	
	*Mrm*-R	AACCACTTGGTCAGGTACAATCACA			
*Lmr*	*Lmr*-F	GTAAATCTTTAGGTAATGTGATTGTAC		690 bp	
	*Lmr*-R	TCTTCTTTAACATGTGGTGTATGAGA			
Molecular mechanisms associated with fusidic acid resistance					
*fusA*	*fusA*-F	TTTACCCTGAGTGTGTTCT	Initial pre-denaturation at 94 °C for 5 min; 30 cycles of denaturation (30 s at 94 °C), annealing (30 s; at 53 °C for *fusB*-*D* and *fusE*, and 40 °C for *fusA*), and extension (2 min, at 72 °C for *fusA*; 50 s, at 72 °C for other genes), followed by a final extension step of 7 min at 72 °C	2250 bp	7
	*fusA*-R	TACATTTAAGCTCACCTTGT			
*fusB*	*fusB*-F	TCATATAGATGACGATATTG		496 bp	26
	*fusB*-R	ACAATGAATGCTATCTCGAC			
*fusC*	*fusC*-F	GATATTGATATCTCGGACTT		128 bp	
	*fusC*-R	AGTTGACTTGATGAAGGTAT			
*fusD*	*fusD*-F	TGCTTATAATTCGGTCAACG		525 bp	
	*fusD*-R	TGGTTACATAATGTGCTATC			
*fusE*	*fusE*-F	CCTAGTGACGTAACAGTAAC		505 bp	
	*fusE*-R	CGGCGWACRTATTCACCTTG			
Molecular mechanisms associated with retapamulin resistance					
*rplC*	*rplC*-F	AACCTGATTTAGTTCCGTCTA	Initial pre-denaturation at 94 °C for 5 min; 30 cycles of denaturation (30 s at 94 °C), annealing (30 s; at 50 °C for *vgaA* and *vgaA*_*LC*_, 52 °C for *vgaB*, *vagC*, *vgaE*, *lsaA*-*C* and *lsaE*, and 55 °C for *rplC*, *cfr*, *vgaAv*, *salA* and 23S RNA V), and extension (2 min, at 72 °C for *vgaC*, *vgaE* and *salA*; 50 s, at 72 °C for other genes), followed by a final extension step of 7 min at 72 °C	822 bp	8
	*rplC*-R	GTTGACGCTTTAATGGGCTTA			
*cfr*	*cfr*-F	GAGATAACAGATCAAGTTTTA		1050 bp	39
	*cfr*-R	CGAGTATATTCATTACCTCAT			
*vgaA*	*vgaA*-F	TCACATGATCGCGCTTTTTTAGAT		770 bp	29
	*vgaA*-R	TCGCTCTCCACCACTTAAGACACT			
*vgaAv*	*vgaAv*-F	CTCTTTGTACGAGTATATGG		770 bp	40
	*vgaAv*-R	GTTTCTTAGTAGCTCGTTGAGC			
*vgaA*_*LC*_	*vgaA*_*LC*_-F	CATTATCGCCATCTGTCA		541 bp	9
	*vgaA*_*LC*_-R	AATTCTTCCGAAGGTTCA			
*vgaB*	*vgaB*-F	TGACAATATGAGTGGTGGTG		577 bp	
	*vgaB*-R	GCGACCATGAAATTGCTCTC			
*vgaC*	*vgaC*-F	CGTATGCCCAGAGTGAG		1073 bp	
	*vgaC*-R	GAGTGCTTCCGTATCCA			
*vgaE*	*vgaE*-F	GAAATATGGGAAATAGAAGATGG		995 bp	
	*vgaE*-R	TGATTCTCTAACCACTCTTC			
*lsaA*	*lsaA*-F	ACCGTGAAGGTGATAAGT		500 bp	
	*lsaA*-R	TTGGGTGTAATCTAACTGAT			
*lsaB*	*lsaB*-F	TCCACTGCCGTTCTTTCC		715 bp	
	*lsaB*-R	AGCCATGTACCGTCCTTT			
*lsaC*	*lsaC*-F	GGCTATGTAAAACCTGTATTTG		429 bp	
	*lsaC*-R	ACTGACAATTTTTCTTCCGT			
*lsaE*	*lsaE*-F	TTGTACGGAATGTATGG		675 bp	
	*lsaE*-R	TTCGCTTCTATTAAGCACTCTT			
*salA*	*salA*-F	CGATGAACCAACAAACCACA		981 bp	10
	*salA*-R	AGGACCGAACCTTGAAATGA			
23S RNA V	23S RNA-F	TGGGCACTGTCTCAACGA		634 bp	41
	23S RNA-R	GGATAGGGACCGAACTGTCTC			
MLST typing					43
*arcC*	*arcC*-F	TTGATTCACCAGCGCGTATTGTC	Initial pre-denaturation at 94 °C for 5 min; 30 cycles of denaturation (30 s at 94 °C), annealing (30 s; at 55 °C for 7 house-keeping genes), and extension (50 s, at 72 °C for other genes), followed by a final extension step of 7 min at 72 °C	456 bp	
	*arcC*-R	AGGTATCTGCTTCAATCAGCG			
*aroE*	*aroE*-F	ATCGGAAATCCTATTTCACATTC		456 bp	
	*aroE*-R	GGTGTTGTATTAATAACGATATC			
*glpF*	*glpF*-F	CTAGGAACTGCAATCTTAATCC		465 bp	
	*glpF*-R	TGGTAAAATCGCATGTCCAATTC			
*gmk*	*gmk*-F	ATCGTTTTATCGGGACCATC		429 bp	
	*gmk*-R	TCATTAACTACAACGTAATCGTA			
*pta*	*pta*-F	GTTAAAATCGTATTACCTGAAGG		474 bp	
	*pta*-R	GACCCTTTTGTTGAAAAGCTTAA			
*tpi*	*tpi*-F	TCGTTCATTCTGAACGTCGTGAA		402 bp	
	*tpi*-R	TTTGCACCTTCTAACAATTGTAC			
*yqiL*	*yqiL*-F	CAGCATACAGGACACCTATTGGC		516 bp	
	*yqiL*-R	CGTTGAGGAATCGATACTGGAAC			

### Pulsed field gel electrophoresis (PFGE)

PFGE was performed for MUP, FA and/or RET resistant strains, as described previously [42]. BioNumerics software 7.0 was used for the analysis of DNA fingerprint profiles. An 80% cutoff value was set to assess the similarity.

### Multilocus sequence typing (MLST)

MLST was conducted by sequencing of the internal fragments of the 7 housekeeping genes, *arcC*, *aroE*, *glpF*, *gmk*, *pta*, *tpi* and *yqiL* on MUP, FA and/or RET-resistant MRSA using the primers (Table 3), as described previously [43]. Sequence types (STs) were determined based on the data from the MLST database for *S. aureus* (http://saureus.mlst.net/).

### Statistical analysis

The difference of MUP, FA and RET-resistant rates of isolates from major samples was analyzed by Pearson’s Chi-square test or Fisher’s exact test using SAS 8.0 (SAS Institute, Cary, NC, USA). Student’s t test was used to determine the difference in the distribution of MIC values between isolates with different mutations. A *P* value < 0.05 (two-tailed) indicated significance.

## Data Availability

All data generated or analyzed during this study are included in this article.

## Abbreviations

FA: Fusidic acid
FAH: High-level fusidic acid resistance
FAL: Low-level fusidic acid resistance
IleS: Isoleucyl-tRNA synthetase
LSH: The Central Hospital of Lishui City, Zhejiang Province
MIC: Minimum inhibitory concentration
MLST: Multilocus sequence typing
MRSA: Methicillin-resistant *Staphylococcus aureus*
MuH: High-level mupirocin resistance
MuL: Low-level mupirocin resistance
MUP: Mupirocin
PCR: Polymerase chain reaction
PFGE: Pulsed-field gel electrophoresis
PT: Shanghai People’s Hospital of Putuo District
RET: Retapamulin
SAH: Shanghai Armed Police Corps Hospital
SGH: Shanghai General Hospital
SSH: Shanghai Sixth People’s Hospital
SGHA: Shuguang Hospital Affiliated to Shanghai University of Traditional Chinese Medicine
